# Waterborne Signaling Primes the Expression of Elicitor-Induced Genes and Buffers the Oxidative Responses in the Brown Alga *Laminaria digitata*

**DOI:** 10.1371/journal.pone.0021475

**Published:** 2011-06-24

**Authors:** François Thomas, Audrey Cosse, Sophie Goulitquer, Stefan Raimund, Pascal Morin, Myriam Valero, Catherine Leblanc, Philippe Potin

**Affiliations:** 1 Marine Plants and Biomolecules Laboratory, Unité Mixte de Recherche 7139, Station Biologique de Roscoff, Université Pierre et Marie Curie, Roscoff, France; 2 Unité Mixte de Recherche 7139, Station Biologique, Centre National de la Recherche Scientifique (CNRS), Roscoff, France; 3 Laboratoire de Biochimie, Epissage, Cancer, Lipides et Apoptose, Unit 613, Institut National de la Santé et de la Recherche Médicale, Faculté de Médecine, Université de Bretagne Occidentale, Brest, France; 4 Adaptation et Diversité en Milieu Marin, Unité Mixte de Recherche 7144, Station Biologique, Université Pierre et Marie Curie, Roscoff, France; 5 Unité Mixte de Recherche 7144, Adaptation et Diversité en Milieu Marin, Station Biologique, Centre National de la Recherche Scientifique (CNRS), Roscoff, France; Biodiversity Insitute of Ontario - University of Guelph, Canada

## Abstract

As marine sessile organisms, seaweeds must respond efficiently to biotic and abiotic challenges in their natural environment to reduce the fitness consequences of wounds and oxidative stress. This study explores the early steps of the defense responses of a large marine brown alga (the tangle kelp *Laminaria digitata*) and investigates its ability to transmit a warning message to neighboring conspecifics. We compared the early responses to elicitation with oligoguluronates in laboratory-grown and harvested wild individuals of *L. digitata*. We followed the release of H_2_O_2_ and the concomitant production of volatile organic compounds. We also monitored the kinetics of expression of defense-related genes following the oxidative burst. Laboratory-grown algae were transplanted in kelp habitats to further evaluate their responses to elicitation after a transient immersion in natural seawater. In addition, a novel conditioning procedure was established to mimic field conditions in the laboratory. Our experiments showed that *L. digitata* integrates waterborne cues present in the kelp bed and/or released from elicited neighboring plants. Indeed, the exposure to elicited conspecifics changes the patterns of oxidative burst and volatile emissions and potentiates this kelp for faster induction of genes specifically regulated in response to oligoguluronates. Thus, waterborne signals shape the elicitor-induced responses of kelps through a yet unknown mechanism reminiscent of priming in land plants.

## Introduction

In land plants, long-distance signaling mediates induced resistance against herbivores and pathogens. The information is not only borne by systemic signals transported in the vascular system, but also by volatile compounds that move in the headspace outside the plant [1] [2]. Among these compounds, green-leaf volatiles and other herbivore-induced volatile organic compounds (VOCs) can mediate the systemic response to local herbivore damage in plants [1] [3] [4]. These VOCs diffuse in the air and potentially also reach neighboring plants, allowing “plant-plant communication", first reported about 25 years ago in trees [5] [6]. Although many ecologists have discounted the possibility of communication between plants [7] [8] [9] [10], recent work demonstrates that numerous taxonomically unrelated plants are capable of eavesdropping, with strong effects on herbivores and plant fitness [11] [12] updated in [13]. It was also proposed that this inter-plant communication is reminiscent of the potentiation of defense responses in animals [14], a so-called primed state that is associated with better or faster induction of the defense response upon biotic or abiotic stress [15].

In the marine environment, exposure to air is intermittent and restricted to intertidal seaweeds. Therefore, waterborne signaling has been hypothesized to represent the counterpart of airborne signaling [16]. Pheromone-mediated mating process is common in the marine environment. During sexual reproduction, most brown algae recognize fatty-acid-derived C8 and C11 hydrocarbons as waterborne sexual pheromones [17]. In the context of biotic interactions, defensive changes can be induced in aquatic prey animals by signals from predators or predator-wounded conspecifics [16]. This phenomenon is especially well documented in freshwater ecosystems [18] [19]. In marine benthic communities, this type of communication has been reported in rockweed (*Ascophyllum nodosum*) — a common brown alga of North Atlantic rocky shores — when it interacts with an herbivorous snail [20] [21] as well as in other species of fucoids challenged with crustacean grazers [22] [23]. Little is known about the chemical structure of these waterborne cues and the steps that lead from their perception to the actual defense response [20], which may express its features only after a secondary attack. Only direct induction of defense responses has been shown to date. In comparison to the current knowledge on the transcriptional responses involved in the defense against pathogens or herbivores in terrestrial plants, changes in gene expression that lead to induced resistance phenomena has only rarely been investigated in marine multicellular algae [24]. Most of the studies on the defense response in marine algae report on the various traits that are expressed *de novo* or at much higher intensities to reduce or prevent further damage, such as oxidative burst-related responses [25] and activation of the synthesis of secondary metabolites [26] [27].

The kelp *Laminaria digitata* belongs to the order Laminariales in brown algae which, together with oomycetes and diatoms, constitute the eukaryotic lineage of Heterokonta or Stramenopiles [28]. Therefore, very distant phylogenetic relationship between brown algae and other eukaryotic lineages, namely metazoans and land plants, raises the possibility that these organisms display distinct defense responses and immunity traits [28]. *L. digitata* recognizes elicitors such as oligosaccharide fragments of alginate (oligoguluronates, GG), its major cell wall component. GGs recognition initiates a cascade of signaling events and leads to an oxidative burst [29] and the control of pathogenic bacteria [30]. At longer term, GGs also induce a resistance against the brown algal epi/endophyte *Laminariocolax tomentosoides* [30]. Lipopolysaccharides (LPS) originating from the outer membrane of Gram-negative bacteria also trigger an oxidative burst in *L. digitata* [31]. Furthermore, polyunsaturated fatty acids and the plant hormone methyl jasmonate lead to resistance to endophytic algae [32]. Cosse *et al.* [33] reported that GGs induce the expression of a set of putative defense genes in *L. digitata*. These genes provide the first markers that can be used to monitor specific gene expression during elicitor-induced defense response in a macroalga. In addition, in response to both biotic (i.e. GG-perception) and abiotic oxidative stress, *L. digitata* naturally emits volatile aldehydes [34] and halocarbons [35]. These compounds are chemically related to VOC species which prime defense responses in terrestrial plants and act as airborne signals [1] [2] [13] [36]. This similarity raises the question of the possible occurrence of distance signaling in kelps.

In this context, this study aims to investigate the ability of challenged kelps to spread a warning message to neighboring conspecifics. First, we compared the responses induced by elicitation in laboratory-grown and freshly harvested or laboratory-acclimated wild algae. This approach showed that the natural environment shapes the elicitor-induced defense responses of *L. digitata*. Hence, we postulated that exposure to waterborne signals from neighboring plants may allow these kelps to prime their defenses and respond more rapidly or perhaps to a greater degree if they are subsequently challenged. To test this hypothesis, we designed two experiments. First, laboratory-grown algae were temporarily reintroduced at a field site in a tide pool colonized by a natural population of *L. digitata*. Furthermore, the effects of this transplantation were mimicked in the laboratory by a novel conditioning procedure based on co-incubation of naive “target" *L. digitata* individuals with “source" individuals that had previously been challenged with GGs (“conditioning pre-treatment") or not (“control pre-treatment"). Here, we address the following questions: (1) do the treatments modify the pattern of oxidative burst in elicited algae, (2) do the conditioned algae respond to GG with an earlier and/or increased expression of defense-related genes; (3) how does conditioning affect the production of VOCs?

## Materials and Methods

### Ethics statement

Relevant permissions were obtained for observational and field studies from the French governmental authorities at Department of Maritime Affairs of Brest.

### Algal material and elicitation procedures

The kelp life cycle consists of a microscopic haploid gametophyte phase, alternating with macroscopic diploid sporophytes. In this study, all experiments were done on the macroscopic diploid individuals. Young *Laminaria digitata* thalli were collected from the field (“wild sporophytes") in two populations separated by 8 km: Pointe Sainte Barbe (+48°43′3564, −3°58′697, Roscoff, Brittany, France) and Ile de Sieck (+48°42′2469, −4°3′5984, Santec Brittany, France). If not used immediately, they were maintained as described in Cosse *et al.* [33] at 14°C with air bubbling in a 10 L flask of filtered seawater (FSW) collected off shore of Roscoff at Astan (+*48*°46′40, −3°56′15), a site with no chemical influence from near shore/intertidal seaweed beds. Laboratory-grown sporophytes were obtained as unialgal cultures grown from random crosses of gametophytes yielded in the laboratory from mature wild sporophytes collected in the same populations. Developing sporophytes were then transferred to larger flasks after 2 wk and grown until they reached a size of about 4 to 6 cm, as previously described [33]. Provasoli Enriched Seawater (PES) culture media prepared with natural FSW from Astan were changed weekly and were illuminated with daylight-type fluorescent lamps at an irradiance of 25 µE.m^−2^.s^−1^ for 10 h per day and kept at 12±1°C.

Alginate oligosaccharides with a polymerization degree ranging from 15 to 25 [37] were prepared in the laboratory by acid hydrolysis according to Haug *et al.* [38] using sodium alginate from *Laminaria hyperborea* stipes (Danisco, Landerneau, France). The purest homopolymeric blocks of poly-alpha-1,4-L-guluronic acid (oligoguluronates, GG blocks) were selected and used as an elicitor at a final concentration of 150 µg.mL^−1^ as described in Küpper *et al.* [29]. During elicitation experiments, hydrogen peroxide concentrations in the seawater were monitored by luminometry as in Küpper *et al.* [29]. Then, 3, 6, and 12 hours after the elicitation, the three replicates were frozen in liquid nitrogen and stored at −80°C prior RNA extraction. These samples were monitored by Reverse Transcription Quantitative PCR (RT-qPCR) for the expression of six previously identified defense-related genes, namely the genes encoding a key enzyme from the pentose phosphate pathway (glucose-6-phosphate dehydrogenase, *g6pd*), two thioredoxins (*trx* and *prx*), two haloperoxidases (*ipo3* and *bpo3*) and a mannitol-1-phosphate dehydrogenase (*mtld*) [33].

### Transient transplantation in the field

The experiments took place at Pointe Sainte Barbe (Roscoff) in November 2007 and April 2008. Six laboratory-grown sporophytes were placed in a 20 L nylon net and transferred into a tide pool, allowing direct contact with the seawater bathing a natural kelp population. Sporophytes were incubated in these conditions for 90 min or 24 h, and taken back to the laboratory with six young thalli of wild sporophytes (4–10 cm in length) harvested from the same tide pool. As control, 6 laboratory-grown sporophytes were introduced into the same tide pool in a sealed transparent 20 L plastic bag filled with filtered seawater (FSW) to prevent contact with natural seawater in the field. Control laboratory-grown sporophytes were kept in FSW in similar bags in culture room at 14°C. All transplanted, wild and control algae were separately reacclimated in laboratory conditions for 24 h. For elicitation experiments, each plantlet was placed separately in a Petri dish (Ø 90 mm) containing 20 mL FSW on a rotary shaker. Three plantlets of each batch were elicited with GG, the three others being kept in FSW. Hydrogen peroxide release was monitored in each Petri dish by luminometry [29]. After three hours of treatment, the plantlets were frozen in liquid nitrogen and stored at −80°C. After RNA extraction, RT-qPCR was used to monitor the expression of the six defense-related genes described in the above section.

### Conditioning procedure in the laboratory

**Figure 1:**
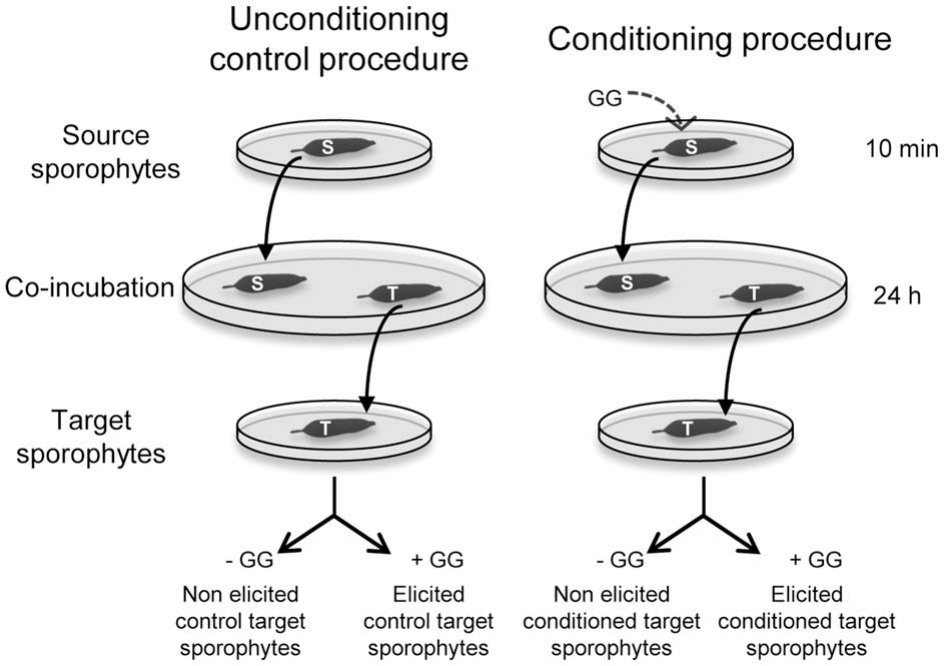
Laboratory pre-treatment procedures to produce conditioned and control algal sporophytes. “Source" sporophytes were either elicited by application of GG in filtered seawater, either handled in the same way without elicitation for control procedure. Control and conditioned “target" sporophytes were co-incubated with non-elicited or elicited source algae, respectively. After 24 hours, the defense responses of each target sporophyte were tested by a subsequent oligoguluronate-elicitation.

Figure 1 shows the detailed design of the laboratory conditioning procedure. Wild sporophytes were harvested at Ile de Sieck and maintained 4 days in a 10 L flask of FSW with air bubbling as described above. For conditioning, “source" sporophytes were elicited by application of GG in FSW for 10 min, and rinsed twice with FSW to remove any traces of elicitors. Control non-elicited source sporophytes were handled in the same way. Control “target" sporophytes (approx. 0.2 to 1 g in weight and 4–10 cm in length) were placed separately in Petri dishes (Ø 140 mm, 150 mL FSW) under agitation together with one non-elicited source sporophyte. Using the same procedure, test target sporophytes were “conditioned" by exposing them to previously elicited source sporophytes. After 24 h of co-incubation, each target sporophyte was transferred to a new Petri dish (Ø 90 mm) for further experiments. Unconditioned and conditioned target sporophytes were elicited separately in 50 mL of FSW, and H_2_O_2_ concentrations were followed by luminometry. FSW was sampled after 1 h to measure VOCs. Experiments were conducted each time with three independent replications. Algal tissues were then frozen in liquid nitrogen after 1.5, 3 and 6 hours and stored at −80°C until RNA extraction. Using RT-qPCR, we measured the relative transcript levels of nine defense-related genes, 5 of the 6 previously measured, namely *g6pd*, *trx*, *prx*, *ipo3*, and *bpo3*, and 4 additional genes, iodoperoxidase 1 (*ipo1*), heat shock protein (*hsp70*), 6-phosphogluconate dehydrogenase (*6pgd2*), and methionine sulfoxide reductase (*msr*), which were also previously shown to be regulated by GG [33].

### Aldehydes and volatile halogenated organic compounds (VHOCs) measurements

Aldehydes were extracted from 25 mL seawater samples and analyzed according to Goulitquer *et al.* [34]. VHOC concentrations in seawater were determined as in Pruvost *et al.* [39] with modifications. VHOCs were separated by purging with a purge-flow of 90 mL.min^−1^ ultra-pure nitrogen for 20 min, focused on a glass bead trap (Grace, DMCS treated, 80/100 mesh) at −120°C and subsequently injected by thermodesorption (100°C, backflush). VHOCs were identified and quantified by comparison with known standard solutions (Ultra Scientific and Supelco).

### RNA extraction and RT-qPCR

Total RNA was extracted using an adapted protocol from Apt *et al.* [40] and treated with Turbo DNase (Ambion, Huntingdon, UK). Total RNA was quantified by Nanodrop ND 1000 spectrophotometer (Labtech International LTD, East Sussex, UK). RT-qPCR was performed as in Cosse *et al.* [33], starting from 400 ng total RNA. Genomic DNA of *L. digitata* was used as reference matrix during each real-time PCR run to generate a standard curve. Results were expressed as number of *L. digitata* genomes per nanogram of total equivalent RNA. Normalization of the transcript levels was performed using a normalization factor defined as the geometric average of the expression of the three reference genes Ld tubulin, Ld actin, and Ld EF1α as recommended in recent published guidelines [41] [42].

### Statistical data analysis

For each defense related gene, statistical differences in the kinetics of expression (time effect) under different conditions (either algal origin or conditioning treatment) were tested by two ways ANOVAs. In the first ANOVA model, we tested for the effects of algal origin (laboratory-grown versus wild sporophytes), time (3, 6 and 12 hours of gene expression time-course) and their interactions on the intensity of gene induction after elicitation by GG. In the second ANOVA model, we tested for the effects of conditioning treatment (conditioned and control algal sporophytes as described in Figure 1), time (1.5, 3 and 6 hours of gene expression time-course) and their interactions, on the transcript levels of defense-related genes. For each ANOVA, the two factors were treated as fixed and Type III sums of squares were used for tests of significance because of the unbalanced design due to one missing value. Indeed, three replicates were generally done for each combination of the two factors except for the first ANOVA, in which only two replicates were done for time = 12 h and origin = lab-grown sporophytes and for the second ANOVA in which only two replicates were done for effects of conditioning treatment = elicited and time = 3 h. General linear model procedures were used. Data were transformed when necessary to meet the assumptions of normality and homogeneity of variance. Multiple comparisons of means were performed using the Tukey-Kramer test method. ANOVAs, multiple comparisons of means, transformation of variables and Student's *t*-test comparison of means were done using MINITAB (version 13.2 MiniTab Inc. 1994, State College USA).

## Results

### Wild and laboratory-grown *L. digitata* sporophytes display different GG-induced responses

**Figure 2:**
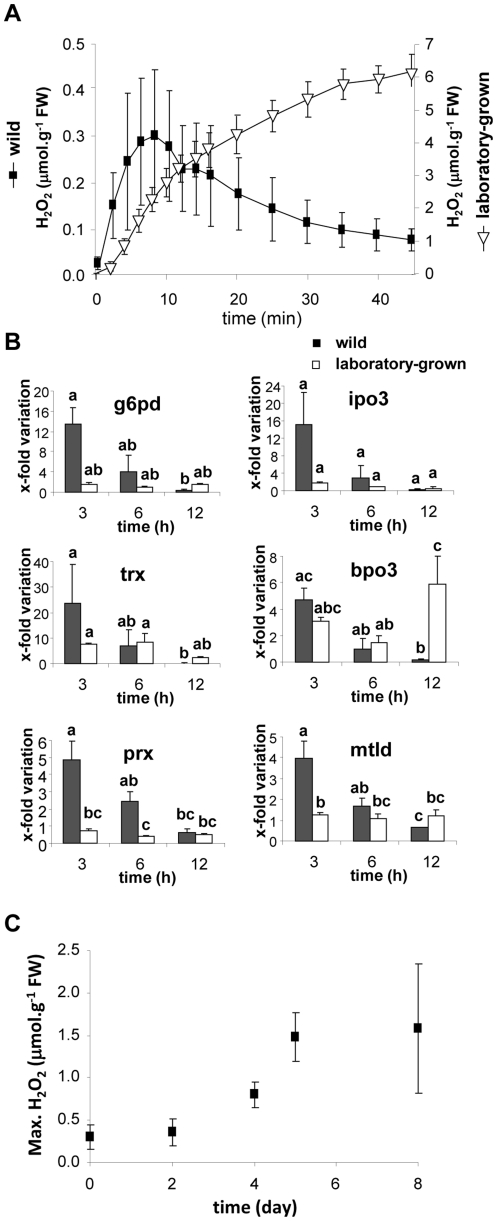
Elicitor-response patterns in laboratory-grown and wild sporophytes of *Laminaria digitata*. **A.** Laboratory-grown (right scale) and harvested wild (left scale) *L. digitata* sporophytes were elicited with oligoguluronates in filtered seawater (FSW) and the concentration of H_2_O_2_ was recorded. Sample size was n = 2–3 thalli and values represent means +/− Standard Errors of Means (SE) on two different scales. **B.** Kinetics of defense-related gene expression in laboratory-grown and harvested wild *L. digitata* sporophytes. Fold variations of transcript levels quantified by RT-qPCR were calculated from different individual thalli (n = 3) between control and elicited sporophytes. For each of the defense related genes, differences between the six conditions (algal origin*time interaction) were tested using Tukey-Kramer test for multiple comparisons of means presented in Table 1 (Letters above the error bars indicate groups that are not significantly different, p<0.05). **C.** Values of the maximum of H_2_O_2_ concentrations reached during the oxidative burst by wild *L. digitata* sporophytes elicited either immediately after harvest from their natural habitats or after laboratory incubation in FSW. Values are mean ± SE (*n* = 3).

To investigate whether containment in a laboratory could modify the defense patterns in a brown alga, we compared the GG-induced responses of laboratory-grown sporophytes of *L. digitata* and freshly collected wild sporophytes of similar size. First, we followed the oxidative response induced by elicitation with GG. In both types of sporophytes, the challenge with GG was rapidly followed by an increase of hydrogen peroxide concentration in the surrounding medium within 10 to 15 minutes. However, the features of the two oxidative bursts were very different according to the origin of the algae (Figure 2A). Laboratory-grown sporophytes released up to 6.21±0.49 µmol.g^−1^ FW of hydrogen peroxide 45 min after elicitation. In comparison, the oxidative burst observed for wild sporophytes was less intense, reaching a maximum of 0.30±0.14 µmol g^−1^ FW of H_2_O_2_ and returning to initial levels within 40 min.

In the same experiment, we profiled the expression kinetics of six previously identified defense-related genes [33]. Statistical analyses revealed a time effect for 5 genes whereas the origin of the algae was a significant factor on *trx* and *prx* gene expression pattern (Table 1). In wild sporophytes, the expression of the six defense marker genes (*g6pd*, *trx*, *prx*, *ipo3*, *bpo3*, *mtld*) was significantly induced and reached maximum levels 3 h after elicitation, returning to the control level within 6 to 12 hours (Figure 2B). In contrast, only *trx* and *bpo3* genes were induced by GGs in laboratory-grown sporophytes and their expression was maximal 6 and 12 h after elicitation, respectively (Figure 2B). The difference of kinetic responses between wild and laboratory grown sporophytes was significant for four defense marker genes (algal origin*time interaction, Table 1).

**Table 1:** Effects of algal origin, time and their interactions on the intensity of gene induction after elicitation by GG.

Factors	genes					
	g6pd	trx	prx	ipo3	bpo3	mtld
**algal origin**	0.605	**0.045**	**0.003**	0.974	0.068	0.128
**time**	**0.017**	**0.005**	**0.030**	0.108	**0.029**	**0.001**
algal origin * time	**0.042**	0.101	**0.025**	0.516	**0.002**	**0.009**

P-values of the two way ANOVAs are given for the six gene expression profiles presented in Fig. 2B. Algal origin: laboratory grown or harvested wild *L. digitata* sporophytes. Time: 3, 6 and 12 hours of gene expression time-course. Significant values are indicated in bold.

We elicited wild *L. digitata* sporophytes collected from the field either immediately, or after 2, 4, 5 and 8 days of incubation in FSW in the laboratory. The longer wild sporophytes were kept in the laboratory, the more intense their oxidative response was (Figure 2C). Four days of incubation in FSW were sufficient to increase the accumulation of H_2_O_2_ by 165%, and it reached 400% after 5 days of incubation.

### Transient transplantation of laboratory-grown *L. digitata* sporophytes in nature modifies subsequent GG-induced responses

**Figure 3:**
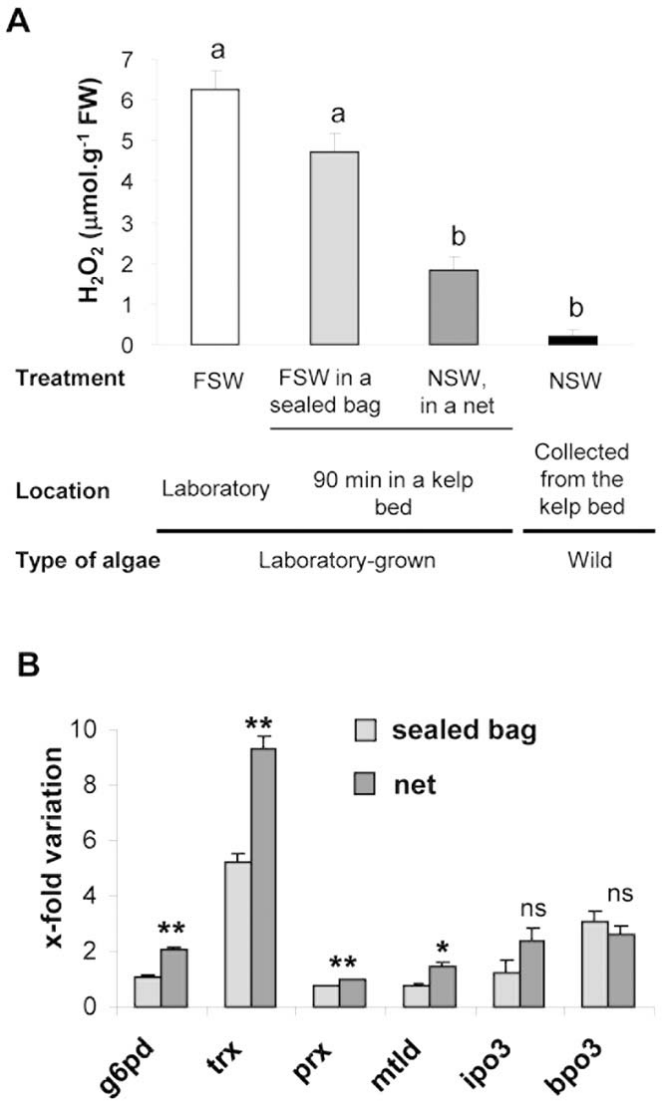
Effects of transplantation to a kelp field of laboratory-grown *L digitata* sporophytes on elicitor-response patterns. Laboratory-grown sporophytes were kept in filtered seawater (FSW) in the laboratory (white) or transferred to a kelp population, either in a hermetically sealed plastic bag filled with FSW (light grey) or in a net allowing direct contact with natural seawater (NSW) (dark grey). Wild sporophytes were harvested from the same kelp bed (black). Sporophytes were taken back to the laboratory and subsequently elicited with GGs. **A.** Values of the maximum amount of H_2_O_2_ detected in FSW after elicitation in laboratory-grown sporophytes, previously transferred (or not) in the kelp bed for 90 min, and wild-type *L. digitata* sporophytes. Values are means ± SE (*n* = 3). Letters above the error bars indicate groups that are not significantly different (Tukey-Kramer test for multiple comparisons of means, p<0.05). **B.** Expression of defense-related genes in laboratory-grown *L. digitata* sporophytes transplanted either in a net or a sealed bag in the kelp bed for 24 hours and subsequently elicited with GGs for 3 h in laboratory. Fold variations of transcript levels quantified by RT-qPCR were calculated between control and elicited sporophytes. Values are means ± SE (*n* = 3). For each defense related genes, differences of fold variations were tested using a *t*-test between algae previously kept in a sealed bag or maintained in a net allowing direct contact with natural seawater (the results of the tests are indicated above the error bars, ns: non-significant, p>0.05; *: p<0.05; **: p<0.01).

The intensity of the oxidative burst of the laboratory-grown sporophytes, field-transplanted in a sealed bag for 90 min, reached 4.71±0.47 µmol H_2_O_2_ g^−1^ FW and was not significantly different from that of algae that had stayed in the laboratory (Figure 3A). In contrast, the sporophytes, field-transplanted in a net, displayed a much less intense oxidative burst (1.84±0.31 µmol H_2_O_2_.g^−1^ FW), which is not significantly different from that observed for wild sporophytes harvested in the same kelp bed (Figure 3A). These experiments were repeated with longer transplantation periods of 24 h with similar patterns of oxidative responses (data not shown). RT-qPCR was used to monitor the expression of defense-related genes 3 h after the GG challenge (Figure 3B). In laboratory-grown *L. digitata* that was transplanted in the field in a sealed plastic bag for 24 h, elicitation induced the expression of only *trx* and *bpo3* after 3 h (5 and 3-fold variations compared to non-elicited control, respectively; Figure 3B). In contrast, for laboratory-grown *L. digitata* was also temporarily transplanted in the field but in a net allowing contact with seawater, the elicitation induced the expression of *g6pd*, *trx*, *mtld*, *ipo3* and *bpo3* (between 1.5 and 9-fold variation compared to non-elicited controls). Moreover, 4 genes (*g6pd*, *trx*, *prx*, *mtld*) showed a significantly different pattern of expression after 3 h of elicitation between algae directly exposed or without contact with natural seawater (Figure 3B).

### Development of a conditioning procedure in the laboratory

**Figure 4:**
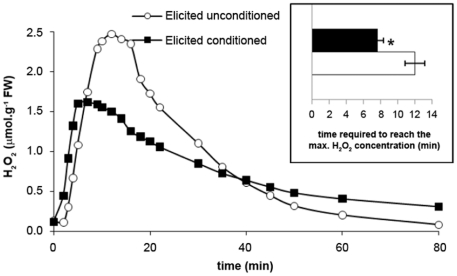
GG-induced oxidative burst in conditioned and unconditioned *L. digitata* sporophytes. Sporophytes were elicited with GGs in seawater and the concentration of H_2_O_2_ was recorded. Experiments were replicated three times and a typical result is shown. **Inset**: Means and standard errors (n = 3) of the time required to reach the maximum of H_2_O_2_ concentrations in the medium after elicitation. The two means were significantly different for conditioned (black bar) and unconditioned (white bar) *L. digitata* sporophytes (*t* test: _*_, P≤0.05).

We developed a novel laboratory assay to further elucidate the phenomenon responsible for the discrepancy observed between wild and laboratory-grown sporophytes and the effect of transplantation in the field (Figure 1). Naive “target" laboratory-grown *L. digitata* sporophytes were co-incubated with “source" laboratory-grown sporophytes that had previously been challenged with GGs or not. Then, target sporophytes were transferred into fresh FSW and further experiments were conducted to characterize their defense responses. Neither the conditioned sporophytes nor the controls constitutively produced extracellular H_2_O_2_ (data not shown). A challenge with GGs triggered an oxidative burst in both conditioned and unconditioned sporophytes (Figure 4). However, maximum H_2_O_2_ accumulation was reached significantly earlier in conditioned sporophytes than in unconditioned ones, after 7.7±0.6 min and 12.0±1.0 min, respectively. External H_2_O_2_ concentrations tended also to be lower in the elicited conditioned sporophytes.

**Figure 5:**
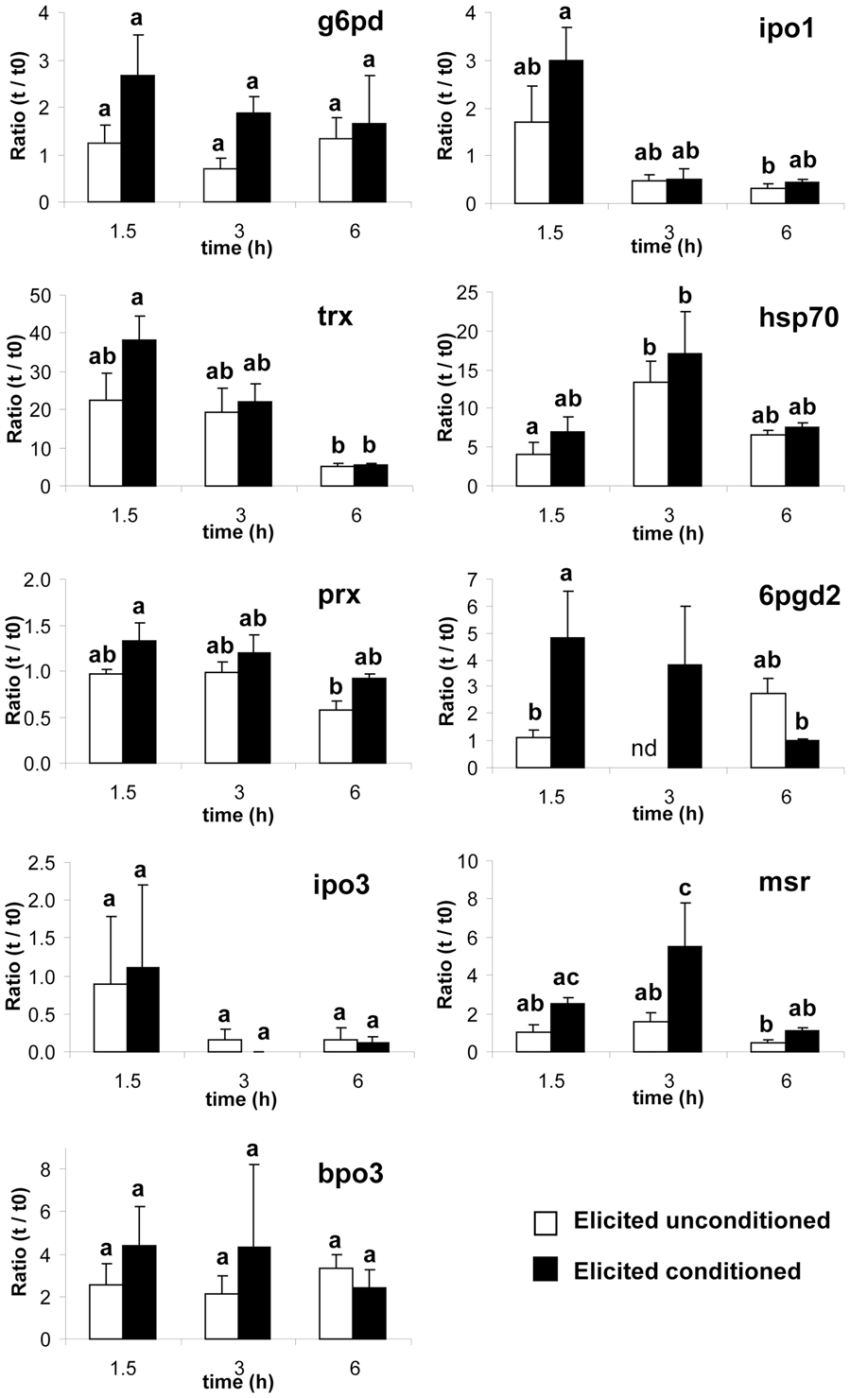
Change in transcript levels of defense-related genes in conditioned and unconditioned *L. digitata* sporophytes after elicitation with GGs. Transcript levels were quantified by RT-qPCR before elicitation (t = 0) and after 1.5 h, 3 h and 6 h. Values represent the fold changes in transcript levels at one time point compared to t = 0 (t/t0, means ± SE, *n* = 3). For each defense related genes, differences between the six conditions (treatment *time interaction) were tested using Tukey-Kramer test for multiple comparisons of means presented in Table 2 (letters above the error bars indicate groups that are not significantly different, p<0.05). For *6pgd2*, statistical analyses were based on two time kinetics (1.5 and 6 h).

Before challenging with GGs, the transcript levels of the nine studied genes were not significantly different in unconditioned and conditioned target sporophytes (*t*-test, P>0.40, *n* = 3, see Table S3). After elicitation, five genes showed a significant regulation over the three time points assessed, two genes (*prx*, *msr*) displayed also a significantly different pattern of expression depending on pre-treatment and for *6pgd2* the interaction between time and pre-treatment was significant (ANOVA, Table 2). When comparing the kinetics of expression pattern, the elicited conditioned algae seem to feature higher levels of induction for almost all the genes (Figure 5). Seven genes out of nine were upregulated in conditioned sporophytes at 1.5 h and down-regulated afterwards (Figure 5). Statistical analyses revealed three main trends for gene regulation. A first one showed no clear up- and down-regulation pattern over the 6 hours, even if genes are induced by GGs, neither significant difference between treatment (*g6pd*, *ipo3* and *bpo3*). A second trend also presented a similar pattern of regulation for both types of algae upon GGs (*trx*, *ipo1* and *hsp70*), but with a rapid (after 1.5 or 3 h), very high and transient up-regulation, especially for *trx* and *hsp70*. A third type of expression pattern showed significant differences between unconditioned and conditioned algae with a earlier (*6pgd2*), faster or stronger (*6pgd2*, *msr*) up-regulation of genes (Figure 5).

**Table 2:** Effects of conditioning treatment, time and their interactions on the intensity of gene induction after elicitation by GG.

Factors	genes								
	g6pd	trx	prx	ipo3	bpo3	ipo1	hsp70	6pgd2*	msr
**treatment**	0.111	0.171	**0.012**	0.989	0.657	0.192	0.165	0.976	**0.002**
**time**	0.589	**0.002**	**0.013**	0.488	0.929	**0.004**	**0.003**	0.141	**0.003**
treatment * time	0.542	0.308	0.835	0.466	0.643	0.67	0.789	**0.003**	0.282

P-values of the two way ANOVAs are given for the nine study genes. Treatments: unconditioned control or conditioning procedures described in Figure 1. Time: 1.5, 3 and 6 hours of gene expression time-course. Significant values are indicated in bold.

*Statistical analyses were based on two time kinetics (1.5 and 6 h).

### The conditioning procedure down-regulates the GG-induced release of VOCs

**Figure 6:**
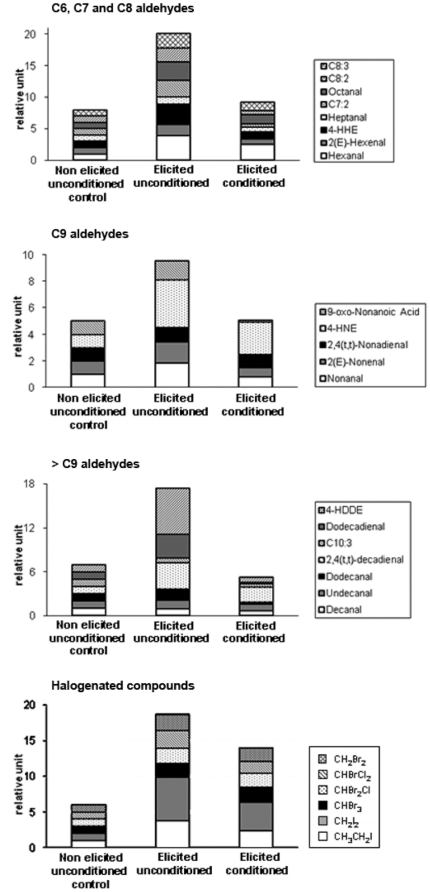
Release of VOCs by conditioned and unconditioned *L. digitata* sporophytes after elicitation with GGs. VOCs were quantified in the medium surrounding *L. digitata* 1 h after challenge or not with GGs. For each compound, the non elicited unconditioned control level was set to a relative unit of 1 to express the fold-variation in the other conditions (absolute concentration values are provided in Tables S1 and S2). 4-HHE, 4-hydroxy-(*E*)-2-hexenal; C7:2, (*E*,*E*)-2,4-heptadienal; C8:2, 2,4-octadienal; C8:3, 2.4.7-octatrienal; 4-HNE, 4-hydroxy-(*E*)-2-nonenal; C10:3, 2.4.7-decatrienal; 4-HDDE, 4-hydroxydodecadienal; CH_3_CH_2_I, iodoethane; CH_2_I_2_, diiodomethane; CHBr_3_, bromoform; CHBr_2_Cl, dibromochloromethane; CHBrCl_2_, bromodichloromethane; CH_2_Br_2_, dibromomethane. Values are means of three independent replicates.

Using this novel conditioning procedure (Figure 1) we monitored the release of volatile organic compounds (VOCs) in the seawater surrounding target sporophytes 1 h after GG elicitation (Tables S1 and S2). Elicitation of unconditioned sporophytes enhanced the emission of most VOCs measured (Figure 6) compared to non-elicited ones. Among aldehydes, the highest fold variations were recorded for 4-HDDE (6-fold increase) and hexanal, 2,4(t,t)-decadienal, dodecadienal, 4-HHE and 4-HNE (3- to 4-fold increases). For the volatile halocarbons, iodoethane (CH_3_CH_2_I) and diiodomethane (CH_2_I_2_) showed the highest increases (6- and 3.7-fold increases, respectively, compared to non-elicited controls). This induction was less pronounced for brominated compounds, the most responsive being bromodichloromethane (CHBrCl_2_) and dibromomethane (CH_2_Br_2_) with a 2-fold increase. In conditioned algae, the 1 h elicitation was not followed by such an increase in the amount of VOCs. The production of most aldehydes by the elicited conditioned sporophytes was equal to or even lower than that measured for non-elicited unconditioned ones. Exceptions were hexanal, 4-HNE and 2,4(t,t)-decadienal and these were only induced 2-fold compared to controls. As aldehydes, the overall elicitation-induced production of halocarbons was also lower in conditioned sporophytes compared to unconditioned sporophytes (Figure 6).

## Discussion

Three main conclusions emerge from our observations and experiments. First, our results show that *L. digitata* sporophytes grown in the laboratory display altered GG-induced responses compared to wild conspecifics freshly harvested in the field. Second, laboratory-grown sporophytes that were transplanted in the field exhibit GG-induced responses that resemble those of wild specimens. This suggests that transient contact with seawater in a kelp beld is sufficient to affect the algal responses to subsequent elicitation with GG. Third, the conditioning procedure that we developed mimics to some extent our field observations. Target sporophytes reacted differently to GG-elicitation according to whether source sporophytes had been elicited or not before co-incubation (conditioning or control procedure, respectively).

Upon elicitation with oligoguluronates, laboratory-grown and wild sporophytes exhibited an oxidative burst, as reported in the literature [29]. However, we showed that H_2_O_2_ levels were 30 times lower in wild sporophytes compared to laboratory-grown specimens (Figure 2A). This more pronounced oxidative burst in laboratory-grown sporophytes was not associated with inter-individual variability as it was also observed in specimens cultured from meiospores isolated from mature sporophytes from populations of Helgoland in Germany [30]. In addition, the gene expression analysis showed that this response cannot be attributed to desensitization of wild sporophytes to GG elicitation: even if their oxidative responses were less intense, wild sporophytes still perceived the defense signal and activated the expression of GG-responsive genes (Figure 2B). Furthermore, our data indicate that this molecular response involves more genes (6 up-regulated genes versus 2) and is more rapid and intense in wild sporophytes than in laboratory-grown specimens (Figure 2B). In terms of kinetics, gene induction in wild algae was consistently and rapidly repressed, returning to initial levels within 12 h after elicitation. In comparison with laboratory-grown algae, the mean induction of expression in wild specimens was higher at 3 h and lower at the end of the experiment. Together, these results support the hypothesis that wild and laboratory-grown *L. digitata* sporophytes are in a different state, which may be explained by their different environmental living conditions, i.e. natural environment vs. controlled culture conditions. This is supported by the fact that the elicitation-induced oxidative responses of wild specimens from the field transferred in culture conditions changed after 5 days, becoming more and more similar to that observed for laboratory-grown sporophytes (Figure 2C).

To investigate the possibility of an effect of the natural environment on the defense capacities of *L. digitata* sporophytes, we conducted transplantation experiments of laboratory-grown algae in a natural kelp population located in a tide pool. We showed that a direct contact with the seawater from the field significantly affects algal responses to subsequent elicitation. Under GG elicitation, transplanted laboratory-grown sporophytes in contact with the seawater displayed a response that resembled that of wild specimens. The oxidative burst was three times less and no more significantly different compared to wild algae (Figure 3A). Moreover four genes instead of two were induced compared to controls maintained in laboratory cultures (Figures 2B and 3B). Algae that were introduced into the same kelp population in a sealed transparent plastic bag to prevent contact with natural seawater in the field did not show this response. The intensity of the elicitation-induced oxidative burst was not significantly different from the non-transplanted controls (Figure 3A). The GG-induced gene response of the specimens transplanted in a sealed bag was also very similar to that of laboratory-grown algae (Figures 2B and 3B). This shows that the observed effect of the natural environment on the defense capacities cannot be attributed to physical parameters such as light or temperature. Indeed, these transplantation experiments suggest that direct contact with natural seawater can explain the discrepancy observed between defense responses of wild and laboratory-grown algae. Significant modification of the laboratory-grown algal responses were obtained only after 90 min of transplantation; we propose that *L. digitata* sporophytes are able to perceive waterborne infochemicals present in the natural environment, enhancing their capacity to efficiently react to further stress.

To test this hypothesis of external defense signals in kelps, we developed a novel experimental assay. Using *L. digitata* sporophytes as sources of potential signals to be perceived by target sporophytes (Figure 1), we showed that target sporophytes react differently to GG elicitation whether source sporophytes had been elicited or not before co-incubation (conditioning or unconditioning control procedure, respectively). Conditioned target sporophytes produced a less intense oxidative burst (Figure 4). This can be explained by a faster triggering of the reactive oxygen species (ROS) detoxification process, because H_2_O_2_ concentration began to decrease significantly earlier in conditioned sporophytes. In addition, as wild specimens in Figure 2B, conditioned sporophytes showed higher and faster up-regulation of genes involved in managing ROS, such as *trx*, *prx* and *msr*, in response to elicitation, compared to unconditioned algae (Figure 5). Before challenging with GGs, the transcript levels were not significantly different in unconditioned and conditioned sporophytes (Table S3). This indicates that the enhanced transcriptional response in conditioned sporophytes is not based on primary induction of defense mechanisms. Despite the limited number of genes tested, the differences are significant for three GG-responsive genes, *msr*, *prx* and *6pgd2* (Table 2). The conditioning procedure has therefore a real effect on subsequent molecular defense responses in *L. digitata*. Altogether, both transplantation and conditioning experiments showed that *L. digitata* integrates waterborne cues present in the kelp bed and/or released from elicited neighboring plants, which later increase reactivity to elicitation.

This is strikingly similar to the priming effect known in the terrestrial environment [43]. In plant cells, this sensitization causes more rapid and/or stronger responses to environmental stresses upon appropriate stimulation. It can be induced biologically by beneficial rhizobacteria and mycorrhizal fungi or through VOCs emitted following plant interactions with pathogens [13] or insects [1]. It is also chemically mediated by application of low doses of salicylic acid (SA), its synthetic analog benzothiadiazole (BTH), jasmonates or ß-aminobutyric acid (BABA) [15] [44] [45]. In *L. digitata* sporophytes, the perception of putative waterborne molecules potentiates the gene response to elicitation (Figure 3). Moreover, conditioned algae displayed faster or stronger elicitation-dependent induction of specific defense genes (Figure 5). These results resemble the priming effects on the expression of defense genes shown in terrestrial plants. In particular, Ton *et al.* [36] found an earlier and/or stronger transcriptional induction of six defense-related genes in maize plants that had previously been in contact with airborne signals from herbivore-infested neighbors. In addition, our results suggest that the priming-like mechanism of *L. digitata* sporophytes affects the way they react to oxidative stress. It has been shown that the oxidative burst is an important prerequisite for induced resistance against a bacterial pathogen [30] and that ROSs may act as signaling agents that trigger defense reactions [33]. However, high levels of ROSs can have deleterious effects on the algal cells if their production and detoxification is not strictly controlled [46]. We suggest that perception of the putative signal potentiates the detoxifying capacities of ROS in primed sporophytes (Figure 4). This would reduce the damage to algal cells while keeping the effect of ROS as toxic compounds against attackers and/or as defense-signaling agents. That the priming-affected genes, such as *prx* and *msr*, are implicated in the oxidative stress management provides further support for this hypothesis.

In response to both biotic and abiotic oxidative stresses, it has been shown that *L. digitata* naturally emits volatile aldehydes [34] and halocarbons [35] in large amounts. The biological significance of distance signaling in conditioned *L. digitata* was further analyzed by monitoring the volatile organic compounds (VOC) released in response to elicitation. We showed that conditioned sporophytes release lower amounts of VOCs in response to GG elicitation compared to unconditioned algae (Figure 6). As VOC emissions depend on oxidative stress in kelps [34], [35], their lower production supports the fact that conditioned algae displayed enhanced ROS detoxification mechanisms.

Taken together, these results indicate that waterborne cues released by neighboring conspecifics shape the responses of kelps to subsequent challenge. These data suggest that priming-like mechanisms exist in kelps and may be a conserved feature of defense and innate immunity among eukaryotic lineages such as brown algae, land plants and mammals, separated by an evolutionary distance of at least 1 billion years [28]. Primed sporophytes show more efficient anti-oxidant responses after elicitation, as shown by H_2_O_2_ (Figures 3A and 4) and VOC (Figure 6) levels, and display faster and/or stronger transcriptional responses (Figure 5). Defense-related waterborne communication in marine algal models has already been reported. Previous studies have demonstrated that external cues released either directly from the brown algae *A. nodosum* and *Fucus vesiculosus* or from feeding grazers were able to directly induce chemical defenses in unharmed conspecifics [20] [22]. However, only late defense responses have been studied so far and only direct induction of defenses has been demonstrated. In the present study, we investigated the earlier steps of the defense responses and showed that waterborne signals also have a potentiating effect, preparing sporophytes to better respond to further challenge without directly triggering defense reactions. It is believed that this priming phenomenon precludes the costly direct allocation of resources to a defense that may eventually not be required, while increasing resistance in case of further attack [47] [48]. In addition to conditioning in the laboratory, the field transplantation experiments we conducted revealed that contact with the natural environment can potentiate the defense responses of *L. digitata*. It confirms that priming mediated by waterborne signals released from *L. digitata*, or potentially from other algae, occurs in nature. This may explain the drastic differences observed for the elicitation-induced oxidative burst (Figure 2A) and transcriptional responses (Figure 2B) of wild algae compared to laboratory-grown sporophytes. The primed state of harvested wild algae is at least partly reversible, as demonstrated by the progressive change in their oxidative response to elicitation after being cultured for a few days in the laboratory. However, even after 8 days of isolation from putative environmental signals in the field, the oxidative response of wild sporophytes does not reach the very high levels of the laboratory-grown algae (Figure 2C). This suggests that the effect of signal perception may persist for longer periods. This observation fits the emerging concept of plant memory or “stress imprint" [49] [50].

Overall, our results demonstrate that waterborne cues induce priming and greatly shape the defense responses of kelps. It raises the question as to the effects at the community level. Most kelp species, including *L. digitata* tend to form highly dense stands that restrict distances between neighboring conspecifics. This proximity allows direct intermittent contacts between blades of the same or of different individuals and might lead to mixing of exudates containing putative signaling compounds. The huge production of biomass in the coastal environment might also provide kelps with a wealth of potential infochemicals. Measurements in tide pools containing *L. digitata* detected the presence of a cocktail of volatile aldehydes [34], alkenes [51] and halogenated compounds [52]. In nature, wild sporophytes are thereby likely to integrate infochemicals to control oxidative burst, production of VOCs and defense-related gene expression. Kelp forests represent both important habitats and food sources for a wide range of consumers and are subjected to multiple biotic (i.e. herbivores, pathogens, etc.) and abiotic stresses (i.e. desiccation, UV, etc.). Previous studies on the *A. nodosum* algal model have shown that waterborne signaling affects the population dynamics of herbivores and predators in controlled laboratory conditions [21] [53]. It has also recently been suggested that resistance to herbivores may be induced in advance by waterborne cues and spread effectively throughout a *F. vesiculosus* belt [23]. In diatoms, perception of sublethal levels of aldehydes such as (2E,4E/Z)-decadienal by cells close to damaged cells could sensitize resistance to successive aldehyde exposure, providing an early-warning protective mechanism, as shown by Vardi *et al.* [54]. In terrestrial plants, priming has been reported to occur in different types of induced resistance and is considered as an important ecological adaptation to environmental stress [4] [13] [48] [55]. Interestingly, it has been shown in *Arabidopsis thaliana* that the fitness costs of priming are lower than those of constitutively activated defenses [47].

Based on these laboratory and field experiments, we hypothesize that inter-individual communication via stress- or defense-related signals may influence the structure of marine communities in coastal ecosystems. The novel conditioning procedure described in this work to prime kelps in the laboratory will facilitate further study of this mechanism, such as the identification of the putative signal(s) and of their impacts on herbivore or pathogen resistance.

## Supporting Information

Table S1 Aldehyde concentrations (ng.mL^−1^.g^−1^ FW) in surrounding seawater before and after a one-hour GG elicitation of *L. digitata* sporophytes. Values are given for three independent replicates.
(DOC)

Table S2 Volatile halocarbon (VHOC) concentrations (pmol.L^−1^.g^−1^ FW) in surrounding seawater before and after a one-hour GG elicitation of *L. digitata* sporophytes. Values are given for three independent replicates.
(PDF)

Table S3 Transcript levels of defense-related genes in conditioned and unconditioned *L. digitata* sporophytes, before elicitation. Values are mean ± s.e.m. (n = 3).
(DOC)
