# Supplementary material for: Waterborne Signaling Primes the Expression of Elicitor-Induced Genes and Buffers the Oxidative Responses in the Brown Alga Laminaria digitata
Source: PLoS One. 2011 Jun 24;6(6):e21475. doi: 10.1371/journal.pone.0021475 (PMC3123347; doi:10.1371/journal.pone.0021475)
Supplement: Table S1 — Aldehyde concentrations (ng.mL−1.g−1 FW) in surrounding seawater before and after a one-hour GG elicitation of L. digitata sporophytes. Values are given for three independent replicates. (DOC) [file pone.0021475.s001.doc]

**Table S1.** Aldehyde concentrations (ng.mL^-1^ .g^-1^ FW) in surrounding seawater before and after a one-hour GG elicitation of *L. digitata* sporophytes. Values are given for three independent replicates.

| **Aldehydes** | **Control** | | | |  | **Elicited unconditioned** | | | |  | **Elicited conditioned** | | | |
| --- | --- | --- | --- | --- | --- | --- | --- | --- | --- | --- | --- | --- | --- | --- |
|  | **#1** | **#2** | **#3** | **mean ± s.e.m** |  | **#1** | **#2** | **#3** | **mean ± s.e.m** |  | **#1** | **#2** | **#3** | **mean ± s.e.m** |
| **Hexanal** | 0.07 | 0.03 | 0.03 | 0.05 ± 0.01 |  | 0.17 | 0.21 | 0.15 | 0.17 ± 0.02 |  | 0.12 | 0.11 | 0.12 | 0.12 ± 0.00 |
| **Heptanal** | 0.03 | 0.01 | 0.13 | 0.06 ± 0.04 |  | 0.02 | 0.07 | 0.11 | 0.07 ± 0.03 |  | 0.04 | 0.05 | 0.04 | 0.04 ± 0.00 |
| **Octanal** | 0.09 | 0.03 | 0.15 | 0.09 ± 0.03 |  | 0.15 | 0.25 | 0.38 | 0.26 ± 0.07 |  | 0.12 | 0.15 | 0.14 | 0.14 ± 0.01 |
| **Nonanal** | 0.41 | 0.29 | 0.11 | 0.27 ± 0.09 |  | 0.30 | 0.54 | 0.63 | 0.49 ± 0.10 |  | 0.19 | 0.17 | 0.26 | 0.21 ± 0.03 |
| **Decanal** | 1.08 | 0.85 | 0.26 | 0.73 ± 0.24 |  | 0.21 | 0.63 | 1.10 | 0.65 ± 0.26 |  | 0.39 | 0.57 | 0.46 | 0.47 ± 0.05 |
| **Undecanal** | 0.24 | 0.21 | 0.05 | 0.17 ± 0.06 |  | 0.16 | 0.19 | 0.23 | 0.19 ± 0.02 |  | 0.21 | 0.08 | 0.16 | 0.15 ± 0.04 |
| **Dodecanal** | 0.08 | 0.51 | 0.16 | 0.25 ± 0.13 |  | 0.28 | 0.37 | 0.54 | 0.40 ± 0.08 |  | 0.06 | 0.05 | 0.10 | 0.07 ± 0.01 |
| **2(E)-Hexenal** | 0.25 | 0.21 | 0.45 | 0.31 ± 0.07 |  | 0.53 | 0.46 | 0.69 | 0.56 ± 0.07 |  | 0.47 | 0.06 | 0.27 | 0.26 ± 0.12 |
| **C7:2** | 0.08 | 0.00 | 0.08 | 0.05 ± 0.03 |  | 0.11 | 0.15 | 0.15 | 0.14 ± 0.01 |  | 0.07 | 0.01 | 0.00 | 0.03 ± 0.02 |
| **C8:2** | 0.03 | 0.05 | 0.04 | 0.04 ± 0.01 |  | 0.09 | 0.10 | 0.08 | 0.09 ± 0.01 |  | 0.06 | 0.01 | 0.01 | 0.03 ± 0.02 |
| **C8:3** | 0.04 | 0.02 | 0.10 | 0.05 ± 0.02 |  | 0.10 | 0.12 | 0.15 | 0.12 ± 0.01 |  | 0.07 | 0.04 | 0.10 | 0.07 ± 0.02 |
| **2(E)-Nonenal** | 0.35 | 0.20 | 0.43 | 0.33 ± 0.07 |  | 0.34 | 0.63 | 0.58 | 0.52 ± 0.09 |  | 0.13 | 0.15 | 0.43 | 0.24 ± 0.10 |
| **2,4(t,t)-Nonadienal** | 0.05 | 0.03 | 0.03 | 0.04 ± 0.01 |  | 0.03 | 0.07 | 0.02 | 0.04 ± 0.01 |  | 0.04 | 0.02 | 0.05 | 0.04 ± 0.01 |
| **2,4(t,t)-decadienal** | 0.09 | 0.06 | 0.02 | 0.06 ± 0.02 |  | 0.12 | 0.18 | 0.30 | 0.20 ± 0.05 |  | 0.08 | 0.04 | 0.21 | 0.11 ± 0.05 |
| **C10:3** | 0.06 | 0.02 | 0.19 | 0.09 ± 0.05 |  | 0.04 | 0.05 | 0.09 | 0.06 ± 0.02 |  | 0.03 | 0.03 | 0.07 | 0.04 ± 0.01 |
| **Dodecadienal** | 0.49 | 0.07 | 0.10 | 0.22 ± 0.13 |  | 0.37 | 1.33 | 0.48 | 0.73 ± 0.30 |  | 0.04 | 0.04 | 0.07 | 0.05 ± 0.01 |
| **4-HHE** | 0.09 | 0.08 | 0.02 | 0.06 ± 0.02 |  | 0.06 | 0.34 | 0.21 | 0.21 ± 0.08 |  | 0.07 | 0.04 | 0.10 | 0.07 ± 0.02 |
| **4-HNE** | 0.53 | 0.40 | 0.42 | 0.45 ± 0.04 |  | 2.25 | 1.95 | 0.65 | 1.62 ± 0.49 |  | 0.54 | 0.08 | 2.69 | 1.10 ± 0.80 |
| **4-HDDE** | 0.10 | 0.37 | 0.12 | 0.20 ± 0.09 |  | 1.81 | 1.67 | 0.27 | 1.25 ± 0.49 |  | 0.09 | 0.10 | 0.23 | 0.14 ± 0.05 |
| **9-oxo-Nonanoic acid** | 0.38 | 0.12 | 0.06 | 0.19 ± 0.10 |  | 0.15 | 0.35 | 0.31 | 0.27 ± 0.06 |  | 0.00 | 0.01 | 0.06 | 0.03 ± 0.02 |
