# Supplementary material for: Waterborne Signaling Primes the Expression of Elicitor-Induced Genes and Buffers the Oxidative Responses in the Brown Alga Laminaria digitata
Source: PLoS One. 2011 Jun 24;6(6):e21475. doi: 10.1371/journal.pone.0021475 (PMC3123347; doi:10.1371/journal.pone.0021475)
Supplement: Table S2 — Volatile halocarbon (VHOC) concentrations (pmol.L−1.g−1 FW) in surrounding seawater before and after a one-hour GG elicitation of L. digitata sporophytes. Values are given for three independent replicates. (PDF) [file pone.0021475.s002.pdf]

Table S2. Volatile halocarbon (VHOC) concentrations ($pmol.L^{-1}.g^{-1}$ FW) in surrounding seawater before and after a one-hour GG elicitation of *L. digitata* sporophytes. Values are given for three independent replicates.

**VHOC**	**Control**				**Elicited unconditioned**				**Elicited conditioned**			
	**#1**	**#2**	**#3**	**mean ± s.e.m**	**#1**	**#2**	**#3**	**mean ± s.e.m**	**#1**	**#2**	**#3**	**mean ± s.e.m**
CH_3CH_2I	24.39	27.88	34.02	28.76 ± 2.81	106.82	68.82	146.73	107.46 ± 22.49	103.10	39.50	65.47	69.36 ± 18.46
CH_2I_2	53.09	41.90	33.39	42.79 ± 5.71	245.03	251.93	284.13	260.36 ± 12.05	136.73	164.09	205.94	168.92 ± 20.12
$CHBr_3$	201.86	359.79	238.13	266.59 ± 47.76	369.95	657.05	579.86	535.62 ± 85.78	530.39	340.15	801.97	557.51 ± 134.00
$CHBr_2Cl$	20.38	27.86	18.37	22.20 ± 2.89	35.88	53.86	48.86	46.20 ± 5.36	42.94	27.94	62.53	44.47 ± 10.02
$CHBrCl_2$	37.64	49.57	20.02	35.74 ± 8.59	92.97	74.65	98.41	88.68 ± 7.19	54.12	54.55	65.22	57.96 ± 3.63
CH_2Br_2	619.20	795.60	586.74	667.18 ± 64.89	1234.15	1207.89	2066.28	1502.77 ± 281.85	1068.32	1286.93	1347.71	1234.32 ± 84.83

CH_3CH_2I, iodoethane; CH_2I_2, diiodomethane; $CHBr_3$, bromoform; $CHBr_2Cl$, dibromochloromethane; $CHBrCl_2$, bromodichloromethane; CH_2Br_2, dibromomethane.
