# Supplementary material for: Waterborne Signaling Primes the Expression of Elicitor-Induced Genes and Buffers the Oxidative Responses in the Brown Alga Laminaria digitata
Source: PLoS One. 2011 Jun 24;6(6):e21475. doi: 10.1371/journal.pone.0021475 (PMC3123347; doi:10.1371/journal.pone.0021475)
Supplement: Table S3 — Transcript levels of defense-related genes in conditioned and unconditioned L. digitata sporophytes, before elicitation. Values are mean ± s.e.m. (n = 3). (DOC) [file pone.0021475.s003.doc]

**Table S3. Transcript levels of defense-related genes in conditioned and unconditioned *L. digitata* sporophytes, before elicitation. Values are mean ± s.e.m. (n = 3).**

|  | **Transcripts level before elicitation** | | | | |
| --- | --- | --- | --- | --- | --- |
|  | **Control**  **pre-treatment** |  | **Conditioning**  **pre-treatment** |  | **Student test**  **p-value** |
| ***trx*** | 0.018 ± 0.009 |  | 0.016 ± 0.009 |  | 0.88 |
| ***prx*** | 0.550 ± 0.099 |  | 0.462 ± 0.037 |  | 0.47 |
| ***g6pd*** | 0.395 ± 0.138 |  | 0.280 ± 0.112 |  | 0.55 |
| ***6pgd*** | 0.069 ± 0.034 |  | 0.048 ± 0.015 |  | 0.88 |
| ***ipo1*** | 0.094 ± 0.033 |  | 0.068 ± 0.037 |  | 0.51 |
| ***bpo1*** | 0.593 ± 0.311 |  | 0.462 ± 0.231 |  | 0.63 |
| ***bpo3*** | 0.034 ± 0.015 |  | 0.022 ± 0.016 |  | 0.43 |
| ***hsp70*** | 0.378 ± 0.118 |  | 0.319 ± 0.190 |  | 0.55 |
| ***gst19*** | 0.028 ± 0.25 |  | 0.012 ± 0.010 |  | 0.90 |
| ***msr*** | 0.010± 0.004 |  | 0.013 ± 0.007 |  | 0.88 |
